# *Oostenbrinkia pedrami* n. sp. (Dorylaimida: Aulolaimoididae) from Iran, with molecular phylogenetic relationships to some other Dorylaimida Pearse, 1942

**DOI:** 10.21307/jofnem-2020-119

**Published:** 2020-11-30

**Authors:** Farahnaz Jahanshahi Afshar

**Affiliations:** Iranian Research Institute of Plant Protection, Agricultural Research, Education and Extension Organization (AREEO), Tehran, Iran

**Keywords:** *Fagus orientalis*, Mazandaran province, Phylogeny, SSU rDNA, Taxonomy

## Abstract

A population of the rarely collected aulolaimoidid genus, *Oostenbrinkia*, was recovered from the rhizosphere soil of *Fagus orientalis* in Mazandaran province, north Iran. *Oostenbrinkia pedrami* n. sp. is mainly characterized by long females, 920 µm with a 6 to 8 µm long odontostyle, 18 to 21 µm long odontophore with well-developed basal ﬂanges, 124 to 187 µm long neck, vulva post equatorial (*V* = 58.5-61.0), and tail short (10-18 µm long, *c*  =  42.5-65.2, *c′* = 0.6-0.9) and rounded. Compared to the generotype species, *O. oostenbrinki*, the new species has a longer odontostyle, total stylet, a posteriorly located vulva, and shorter rounded tail, and compared to the only one other representative, *O. parva*, longer females with longer odontostyle and total stylet. In molecular phylogenetic analyses using a near-full-length sequence of the small subunit ribosomal DNA (SSU rDNA), the new species appeared as an independent basal lineage to the included ingroup species. This is the first molecular data of this family.

The nematode family Aulolaimoididae (Jairajpuri, 1964), currently placed in the dorylaimid superfamily Tylencholaimoidea (Filipjev, 1934), is mainly characterized by the presence of a basket-like structure in the stoma formed of small ribs, tripartite pharynx, and a distinct muscular pharyngeal bulb with a thickened valvular chamber. It represents a group of rarely collected soil-inhabiting nematodes (Andrássy, 2009). According to Mushtaq et al. (2006), this family contains 15 valid species belonging to four genera: *Aulolaimoides* (Micoletzky, 1915), *Adenolaimus* (Andrássy, 1973), *Oostenbrinkia* (Ali et al., 1973), and *Cladocephalus* (Swart and Heyns, 1991). The study by Mushtaq et al. (2006) and Pedram et al. (2012) represents the most recent studies on this rarely encountered family group.

The genus *Oostenbrinkia* is characterized by its smooth lip region, nonprotruding labial papillae, didelphic-amphidelphic genital system, and a short, rounded tail. It currently harbors two species, *O. oostenbrinki* (Ali et al., 1973), a large species (*L* = 780, 840 μm), and *O. parva* (Mushtaq et al., 2006) a smaller form (*L* = 440, 490 μm). Until now, only two species of the family Aulolaimoididae, *Adenolaimus tropicus* (Mushtaq et al., 2006) and *Oostenbrinkia parva*, have been recorded from Iran (Pedram et al., 2012); they added new information for the two aforementioned species.

During the present study, a population of an unknown species of the rarely collected genus *Oostenbrinkia* was recovered from natural forests of Mazandaran province in north Iran. The comparisons with two currently valid species under the genus revealed that it belongs to an unknown species, described herein as *Oostenbrinkia pedrami* n. sp. The molecular phylogenetic analysis for the new species, as the first representative of the family, was also performed.

## Materials and methods

### Sampling, nematode extraction, mounting, and drawing

Several soil samples were collected from natural forests and grasslands of Mazandaran province during July to August 2019. Nematodes were extracted from soil samples by means of the tray method (Whitehead and Hemming, 1965). Nematodes were observed and handpicked directly under a Nikon SMZ1000 stereomicroscope. The specimens were killed with hot 4% formaldehyde solution, transferred to anhydrous glycerin according to De Grisse (1969), and mounted on permanent slides. The specimens were examined using a Nikon Eclipse E600 light microscope. Photomicrographs were taken using an Olympus DP72 digital camera attached to an Olympus BX51 microscope equipped with differential interference contrast. Drawings were made using a drawing tube attached to the microscope and were redrawn using the CorelDRAW® software version 2017.

### DNA extraction, PCR, and sequencing

For DNA extraction, a live individual nematode of the collected population of *Oostenbrinkia* was picked out, washed using distilled water, observed on a temporary slide, photographed, transferred to a small drop of Tris-EDTA (TE) buffer (10 mM Tris-Cl, 0.5 mM EDTA, pH 9.0, Qiagen) on a clean slide, and squashed using a clean slide cover with the aid of a pipette tip. The suspension was collected by adding 20 μl of TE buffer after gently removing the slide cover and leaving the solution on the slide (Pedram, 2017). The DNA sample was stored at −20°C until used as the polymerase chain reaction (PCR) template. The near-full-length sequence of the small subunit ribosomal DNA (SSU rDNA) was ampliﬁed using the forward primer 18S4 (5′-GCTTGTCTCAAAGATTAAGCC-3′) (Blaxter et al., 1998) and the reverse primr 18S-1573R (5′-TACAAAGGGCAGGGACGTAAT-3′) (Mullin et al., 2005). The PCR reaction for the amplification was performed according to Pedram (2019). The newly obtained sequence was deposited into the GenBank database under the accession number MT860062.

### Phylogenetic analyses

The raw file of the newly generated sequence of SSU rDNA of *Oostenbrinkia pedrami* n. sp. was manually checked, edited, and compared with several other sequences of dorylaimid species available in GenBank database using the BLAST homology search program. The 30 selected sequences (maximal number of available SSU sequences of Tylencholaimoidea and sequences from other dorylaimid genera) were aligned with the Q-INS-i algorithm of the online version of MAFFT version 7 (http://mafft.cbrc.jp/alignment/server/) (Katoh and Standley, 2013). The online version of Gblocks 0.91b (Castresana, 2000) was used to eliminate ambiguous parts of the alignment, with all three options for a less-stringent selection (http://molevol.cmima.csic.es/castresana/Gblocks_server.html). The most appro-priate substitution model for the dataset was selected using the Akaike information criterion (AIC) by using PAUP^∗^/MrModeltest v2 (Nylander, 2004). A general time-reversible model, including a gamma distribution for rates across sites and a proportion of invariant sites (GTR + G + I), was selected for the phylogenetic analysis of the SSU dataset. Bayesian inference (BI) was performed using MrBayes v3.1.2 (Ronquist and Huelsenbeck, 2003) with selecting a random starting tree and running the chains for 3 × 10^6^ generations. After discarding burn-in samples, the remaining samples were retained for further analyses. The Markov chain Monte Carlo (MCMC) method within a Bayesian framework was used to estimate the posterior probabilities of the phylogenetic trees (Larget and Simon, 1999) using the 50% majority rule. The resultant phylogenetic tree was visualized with Dendroscope V.3.2.8 (Huson and Scornavacca, 2012) and drawn in CorelDRAW software version 16.

## Results

### Systematics

**Figure 1:**
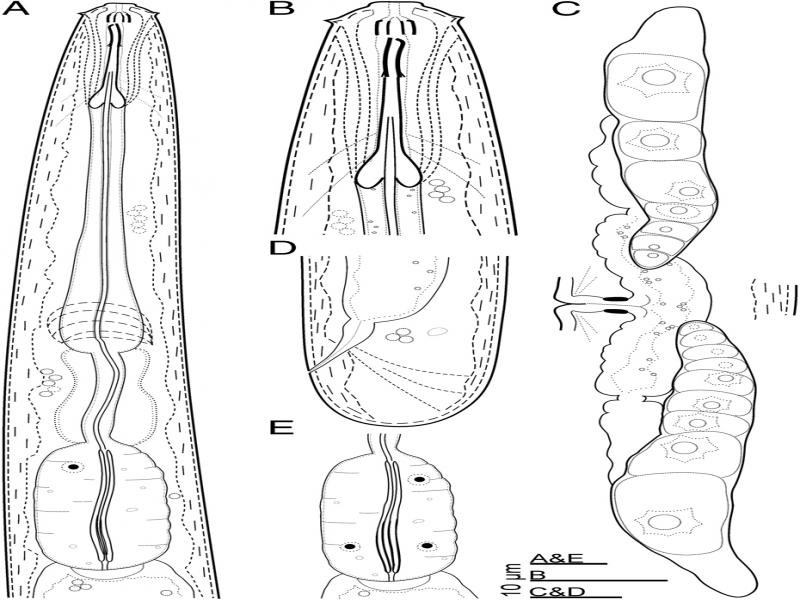
Line drawings of *Oostenbrinkia pedrami* n. sp., female. (A): Neck region; (B): Anterior end; (C): Reproductive system; (D): Posterior body region; (E): Pharyngeal bulb with triquetrous chamber in its lumen.

**Figure 2:**
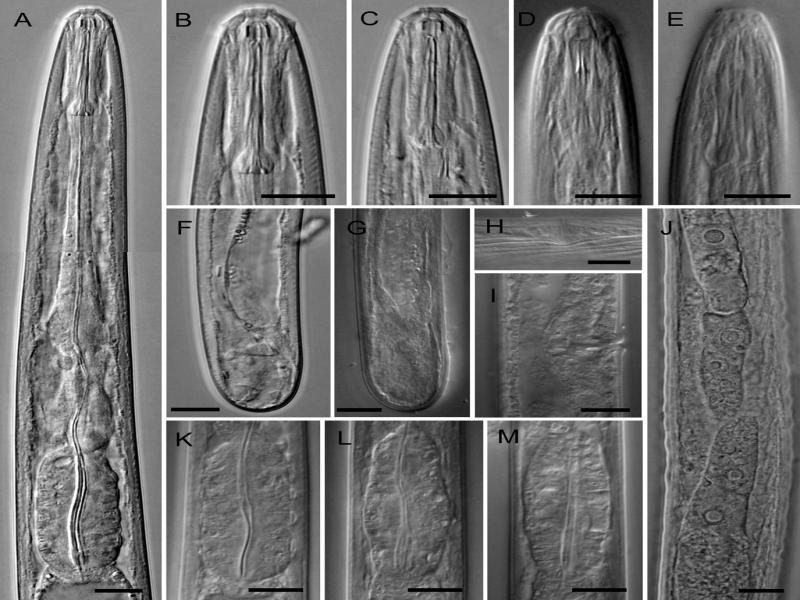
Light photomicrographs of *Oostenbrinkia pedrami* n. sp., female and juvenile (A-C, F: Fresh sample in water; C, Juvenile). (A): Neck region; (B, D, E): Anterior body region in female; (C) Anterior body region; (F, G): Posterior body region; (H, I): Vulva region; (J): Part of the reproductive system showing reflexed ovaries; (K-M): Pharyngeal bulb with variation of triquetrous chamber in its lumen. (Scale bars = 10 µm).

*Oostenbrinkia pedrami* n. sp. (Figs. 1, 2).

### Measurements

Measurements of the new species are given in Table 1.

**Table 1.** Morphometrics of *Oostenbrinkia pedrami* n. sp.

Character	Holotype	Paratypes
*n*	–	10
*L*	847	747 ± 83 (648-920)
*a*	27.3	31.0 ± 1.8 (27.3-33.5)
*b*	5.0	4.7 ± 0.4 (4.0-5.5)
*c*	65.2	56.8 ± 8.8 (42.5-65.2)
*c′*	0.7	0.8 ± 0.1 (0.6-0.9)
*V*	59.0	59.7 ± 0.9 (58.5-61.0)
Lip region width	10	9.7 ± 0.4 (9.0-10.5)
Lip region height	5	4.6 ± 0.5 (4-5)
Odontostyle	6.5	7.0 ± 0.6 (6-8)
Odontophore	19.5	19.6 ± 1.2 (18-21)
Total stylet length	26	26.7 ± 1.6 (24-29)
Anterior end to nerve ring	98	93.1 ± 5.2 (87-102)
Neck length	171	159.4 ± 17.5 (124-187)
Body width at mid-body	31	24.3 ± 3.9 (20-31)
Body width at anus/cloaca	20	17.8 ± 1.6 (15.5-20.0)
Body width at the base of terminal bulb	25.5	22.0 ± 1.9 (19.0-25.5)
Anterior genital tract	109	70.4 ± 21.3 (45-109)
Posterior genital tract	115	74 ± 22 (52-115)
Length of terminal bulb	36	39.1 ± 2.0 (36.0-42.5)
Width of terminal bulb	17	14.7 ± 2.0 (12-17)
Flanges width	4	4.9 ± 0.6 (4-6)
Anterior genital branch as % to L (G1)	12.9	9.5 ± 2.3 (6.4-12.9)
Posterior genital branch as % to L (G2)	13.6	10.0 ± 2.2 (7.3-13.6)
Prerectum/anal body width	5.5	4.6 ± 1.3 (2.6-6.0)
Cardia length	6.5	5.7 ± 0.8 (5-7)
Cardia width	9.5	9.4 ± 0.4 (9-10)
Anterior end to vulva	495.5	434 ± 32 (395-495)
Prerectum length	110	97.4 ± 26.0 (50-125)
Rectum length	14	12.6 ± 2.0 (10-16)
Tail length	13	13.6 ± 2.4 (10-18)

**Note:** All measurements are in μm and in the form: mean ± standard deviation (range).

### Female

The body was moderately long, cylindrical, gradually tapering anteriorly, and straight to slightly ventrally curved when relaxed. The cuticle was smooth, thin, 1.0 µm thick at mid-body, and 1.5 µm thick on the tail, not thickened on the tail tip. The lateral chords were 5.5 to 8.5 µm wide or 26.2 to 37.5% of body diameter at mid-body. Lip region was continuous with body contour, appearing angular due to the prominent outer labial papillae (Figs. 1B, 2B), *ca.* twice as wide as high and 39.2 to 50.0% as wide as body diameter at neck base; perioral disk-like differentiation was prominent, but the cephalic papillae were small. Amphidial fovea was cup-shaped, but its aperture was a hardly observed small slit. The cheilostom was wide, including distinctly refractive ribs forming a basket-like structure (Figs. 1B, 2B). The odontostyle was robust, with distinct lumen and aperture, 5.0 to 6.5 times as long as wide, 1.2 to 1.7 times shorter than lip region diameter, and somewhat irregular in shape with the dorsal side slightly sigmoid and the anterior end apparently wider (Fig. 1B). The odontophore was about three times as long as the odontostyle, with basal flanges. The guiding ring was indistinct. The pharynx consisted of three sections: an anterior slender part 64 to 77 µm long, occupying 38 to 43% of the total neck length, encircled by the nerve ring at the swollen posterior end, middle isthmus-like part 16 to 30 µm long, 10 to 18% of the total neck length, surrounded by glandular tissue, and posterior basal bulb, 37 to 46 µm long, 23 to 25% of the total neck length, with a triquetrous chamber in posterior half with thickened walls well observed in some specimens (Fig. 2K-M), and three pharyngeal gland nuclei seen: one dorsal, two ventromedian. The cardia hemispheroid wider than long. The genital system was didelphic-amphidelphic, and both branches were short and of equal length. The ovaries reflexed, usually reaching the vulva, the oocytes in a single row except in the germinal zone, the oviduct connecting the tubular uterus with the sphincter, vagina less than half-corresponding body width long, *pars distalis vaginae* about 5 µm long, *pars refringens vaginae* apparently present, as sclerotized pieces about 3 µm long and *pars proximalis vaginae* small (Figs. 1C, 2I), and vulva a transverse slit, post equatorial. Prerectum length was 2.6 to 6.0 times the anal body diameter, rectum length was about equal to anal body diameter. The tail was short and rounded.

### Male

Not found.

### Type host and locality

Rhizosphere soil of *Fagus orientalis*, was collected in the forests of Shahsavar, Mazandaran province, north Iran. GPS coordinates are N 36°40′1.401″, E 50°49′9.48″, elevation 678 m.

### Type specimens

The holotype female and nine paratype females (slide accession codes TM5087-TM5090) are deposited in the Nematode Collection of Faculty of Agriculture, Tarbiat Modares University, Tehran, Iran. The LSID code of this publication is urn:lsid:zoobank.org:pub:7E69C867-1C83-4260-A64E-2C87D06D14F6.

### Etymology

The new species is named in honor of Dr. M. Pedram, the well-known Iranian nematologist.

### Differential diagnosis

*Oostenbrinkia pedrami* n. sp. is mainly characterized by its large body size, 747 (648-920) µm long, the lip region appeared continuous with body contour, appearing angular due to the prominent outer labial papillae, a well-developed disk-like differentiation in the frontal end, a distinct basket-like structure at anterior cheilostom, 7 (6-8) µm long, odontostyle slightly wider at the anterior end, 27 (24-29) µm long total stylet, vulva post equatorial (*V* = 60 (58.5-61.0)), and a short, rounded tail (*c* = 57 (42.5-65.0), *cʹ* = 0.8 (0.6-0.9)). The comparisons of the new species with two currently known species of the genus are given as follows:

From *O. oostenbrinki*, by a longer odontostyle (7 (6-8) vs 5 µm), longer total stylet (26.7 (24-29) vs 20 µm), posterior position of vulva (*V* = 60 (58.5-61.0) vs 49-50), and a tail rounded and shorter with the *c* equal to 0.8 (0.6-0.9) (vs cylindrical, slightly tapering posteriorly and *c* equal to 2.7).

From *O. parva* (the morphometric data ranges after the type and the second Iranian population by Pedram et al. (2012)) by longer females (747 (648-920) vs 390-530 µm), longer odontostyle (7 (6-8) vs 4 µm), and total stylet (27 (24-29) vs 16-20 µm).

### Molecular profiles and phylogenetic status

**Figure 3:**
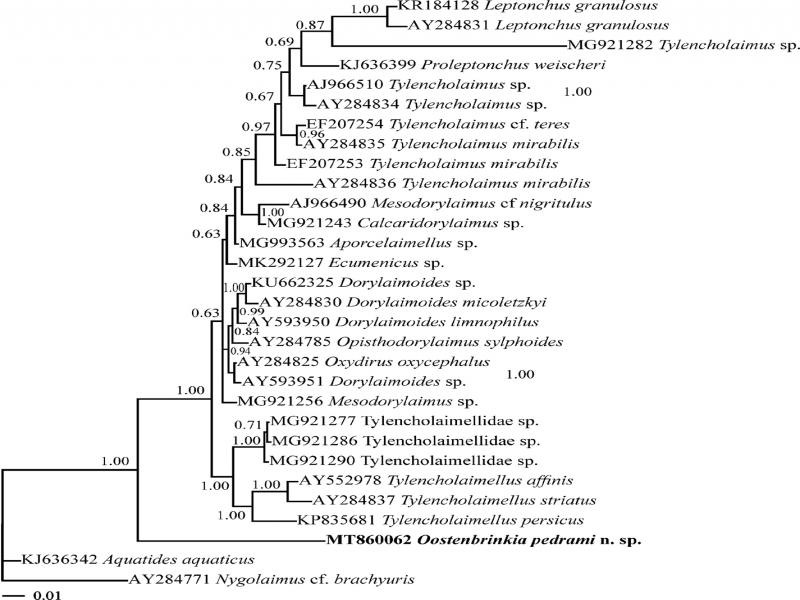
Bayesian 50% majority rule consensus tree of *Oostenbrinkia pedrami* n. sp. based on SSU rDNA sequences under GTR + I + G model. Bayesian posterior probability values more than 0.50 are given for appropriate clades. The new sequence is indicated in bold.

A 1,562 nucleotide long sequence from *Oostenbrinkia pedrami* n. sp. (MT860062) was used to infer the SSU phylogeny together with 28 sequences of Tylencholaimoidea and other assorted Dorylaimida (Pearse, 1942). Sequences from two species of Nygolaimidae (Ahmad and Jairajpuri, 1979) from suborder Nygolaimina (Ahmad and Jairajpuri, 1979), the sister group of Dorylaimina (Pearse, 1936) in SSU phylogeny, as resolved by Mullin et al. (2005), served as outgroup taxa (species names and accession numbers in Fig. 3). The SSU rDNA dataset was composed of 1,704 characters, of which 309 were variable. A Bayesian phylogenetic tree was inferred from this dataset (Fig. 3). *Oostenbrinkia pedrami* n. sp. appeared as an independent lineage in a basal position, as the sister clade to all included ingroup taxa in this tree.

## Discussion

Aulolaimoididae currently contains 15 species belonging to four genera (Goseco et al., 1975; Swart and Heyns, 1991; Mushtaq et al., 2006). Three species are now known from Iran (Pedram et al., 2012, this paper).

There is a paucity of dorylaimid sequences in GenBank; consequently, the phylogeny of the family is not well known. In the presently resolved SSU phylogeny, the new species occupied a basal position in the tree, as an independent lineage, and only distantly related to other Tylencholaimoidea. The unusual stylet shape and pharyngeal structure do suggest a congruence with the molecular results. Nevertheless, this basal position needs to be tested with additional species of Aulolaimoididae before it can be accepted as reflecting Dorylaimida phylogeny. Collectors of aulolaimoidid specimens should make it a priority to reserve some for molecular analysis.
